# Daily Early-Life Exposures to Diet Soda and Aspartame Are Associated with Autism in Males: A Case-Control Study

**DOI:** 10.3390/nu15173772

**Published:** 2023-08-29

**Authors:** Sharon Parten Fowler, David Gimeno Ruiz de Porras, Michael D. Swartz, Paula Stigler Granados, Lynne Parsons Heilbrun, Raymond F. Palmer

**Affiliations:** 1 Department of Medicine, Joe R. & Teresa Lozano Long School of Medicine, The University of Texas Health Science Center at San Antonio, 7703 Floyd Curl Drive, San Antonio, TX 78229, USA; 2 Southwest Center for Occupational and Environmental Health, Department of Epidemiology, Human Genetics and Environmental Sciences, School of Public Health in San Antonio, The University of Texas Health Science Center at Houston, 7411 John Smith Drive, San Antonio, TX 78229, USA; david.gimeno@uth.tmc.edu; 3 Center for Research in Occupational Health, Universitat Pompeu Fabra, 08003 Barcelona, Spain; 4 CIBER of Epidemiology and Public Health, 28029 Madrid, Spain; 5 Department of Biostatistics and Data Science, School of Public Health, The University of Texas Health Science Center at Houston, 1200 Pressler Street, Houston, TX 77030, USA; michael.d.swartz@uth.tmc.edu; 6 Divisions of Environmental Health and Global Health, School of Public Health, San Diego State University, 5500 Campanile Drive, San Diego, CA 92182, USA; pstiglergranados@sdsu.edu; 7 Department of Epidemiology, Human Genetics, and Environmental Sciences, School of Public Health in San Antonio, The University of Texas Health Science Center at Houston, 7411 John Smith Drive, San Antonio, TX 78229, USA; lynne.heilbrun@uth.tmc.edu; 8 Department of Family Practice and Community Medicine, Joe R. & Teresa Lozano Long School of Medicine, The University of Texas Health Science Center at San Antonio, 7703 Floyd Curl Drive, San Antonio, TX 78229, USA; palmerr@uthscsa.edu

**Keywords:** autism, autism spectrum disorder, pregnancy, diet, diet soda, diet beverages, aspartame, non-nutritive sweeteners, artificial sweeteners, high-intensity sweeteners

## Abstract

Since its introduction, aspartame—the leading sweetener in U.S. diet sodas (DS)—has been reported to cause neurological problems in some users. In prospective studies, the offspring of mothers who consumed diet sodas/beverages (DSB) daily during pregnancy experienced increased health problems. We hypothesized that gestational/early-life exposure to ≥1 DS/day (DS_early_) or equivalent aspartame (ASP_early_: ≥177 mg/day) increases autism risk. The case-control Autism Tooth Fairy Study obtained retrospective dietary recalls for DSB and aspartame consumption during pregnancy/breastfeeding from the mothers of 235 offspring with autism spectrum disorder (ASD: cases) and 121 neurotypically developing offspring (controls). The exposure odds ratios (ORs) for DS_early_ and ASP_early_ were computed for autism, ASD, and the non-regressive conditions of each. Among males, the DS_early_ odds were tripled for autism (OR = 3.1; 95% CI: 1.02, 9.7) and non-regressive autism (OR = 3.5; 95% CI: 1.1, 11.1); the ASP_early_ odds were even higher: OR = 3.4 (95% CI: 1.1, 10.4) and 3.7 (95% CI: 1.2, 11.8), respectively (*p* < 0.05 for each). The ORs for non-regressive ASD in males were almost tripled but were not statistically significant: DS_early_ OR = 2.7 (95% CI: 0.9, 8.4); ASP_early_ OR = 2.9 (95% CI: 0.9, 8.8). No statistically significant associations were found in females. Our findings contribute to the growing literature raising concerns about potential offspring harm from maternal DSB/aspartame intake in pregnancy.

## 1. Introduction

Over the past 40 years, the prevalence of diagnosed autism spectrum disorder (ASD) in the U.S. has dramatically risen [1], from fewer than 0.3 per 1000 children diagnosed with autism before 1980 [2] to 27.6 per 1000 children diagnosed with ASD in 2020 [3]. Changes in diagnostic definitions and guidelines and increased testing availability and funding have made major contributions to this increase in diagnosed cases; under the added impacts of changes in dietary, environmental, and other exposures affecting the intrauterine environment, ASD prevalence has reached unprecedented proportions. Males have been disproportionately affected: autism prevalence among boys is almost quadruple that among girls, and a recent study estimated that approximately 1 in 23 U.S. boys aged 8 years or older in 2020 had been diagnosed with ASD [3]. The degree to which ASD diagnoses have risen during this time highlights the potential role of non-genetic influences, including early prenatal exposures to heavy metals, organophosphate pesticides, and other environmental toxins, in offspring autism risk [4].

Maternal diet during pregnancy and breastfeeding represents an important additional non-genetic influence on offspring autism risk and has been studied increasingly closely over the past 15 years. Zhong et al. [5], in their systematic review, reported evidence of the protective role of maternal intake of prenatal vitamins, folic acid, and vitamin D in reducing offspring autism risk. Peretti et al. [6] also reported decreased offspring autism risk associated with a higher maternal intake of folic acid, omega 6 fatty acids, and vitamin D during pregnancy and an increased autism risk among the offspring of mothers deficient in omega 3 fatty acids and polyunsaturated fatty acids. Although the results from different individual studies varied, the available data suggest that these selected nutrients may indeed exert neuroprotective effects during development.

In contrast, the maternal dietary intake of methanol during pregnancy was identified as a potential risk-increasing exposure for offspring. Peretti et al. [6] called attention to the work of Walton and Monte [7], who found that the dietary intake of methanol during pregnancy was approximately twice as high among biological mothers of children with ASD as among mothers of neurotypically developing children. Aspartame, a leading non-nutritive sweetener (NNS) in the U.S., is a ubiquitous source of dietary methanol in this country, and aspartame-sweetened products constituted the major sources of dietary methanol included in Walton and Monte’s calculations of maternal methanol intake. If their preliminary findings are correct, the possibility that pregnant women might unknowingly be exposing their unborn children to an increased autism risk through their consumption of aspartame-sweetened diet products during pregnancy is of particular concern.

The safety of aspartame, a leading sweetener used in diet sodas, other diet beverages, and over 6000 dietary, pharmacologic, and other products over the past 40 years [8], has been the subject of intense debate since before it entered the marketplace. In 1981, the U.S. Food and Drug Administration (FDA) approved the use of aspartame as a tabletop sweetener and, in 1983, as an ingredient in diet sodas (DS) and other products [8]. Aspartame rapidly became the leading DS sweetener used in the U.S; DS consumption doubled, and aspartame consumption increased 17-fold, over the next 8 years [9]. By the end of 1983, however, the FDA had received numerous complaints of adverse reactions among aspartame consumers. Prominent complaints included headache, anxiety, and depression [10,11]. These and other neurological problems—including irritability, mood disorders, cognitive problems, and seizures—were subsequently reported to be increased among users of diet sodas/beverages (DSB) and other aspartame-sweetened products [10,11,12,13,14,15,16,17,18,19,20,21,22], which are leading vehicles of aspartame intake in the U.S. [23]. Impaired cognitive performance and increased anxiety-related behaviors also developed in aspartame-fed animals [24,25,26,27,28,29,30]. Such reports contributed to the prolonged controversy over the safety of aspartame [29,31,32,33,34,35,36,37]. While a review of this debate is beyond the scope of this report, we highlighted results with potential neurophysiologic relevance to our investigation.

Each of the three first-phase metabolites of aspartame has been studied with regard to its impacts on neurologic function. Aspartame is metabolized in the intestine into aspartic acid, an excitatory neurotransmitter; phenylalanine, which is involved in neurotransmitter regulation; and methanol, the metabolites of which include formaldehyde, formate, and other toxins [38]. These three first-phase metabolites represent approximately 40%, 50%, and 10% of aspartame by weight, respectively [38]. The adverse neurological impacts following the consumption of aspartic acid, phenylalanine, and/or aspartame include changes in neurotransmitter levels [25,38,39] and excitotoxicity, with adverse impacts on neuron function/survival [40,41,42]. Primates, including humans, are uniquely vulnerable to methanol [43,44], the blood levels of which rise following aspartame consumption [44,45,46]. Exposures to methanol and formaldehyde resulted in increased neuronal apoptosis, neurodegeneration, and cognitive problems [43,47,48].

The potential mechanisms that might underlie the associations between aspartame intake and long-term health problems in humans and their offspring were reported in animal studies. Prominent among these is a recurring finding of the markedly decreased availability of the reduced form of glutathione—glutamine sulfhydryl (GSH)—in aspartame-fed animals [25,44,45,46,49,50,51,52]. This finding is crucial because GSH protects the developing brain by providing antioxidant defense against oxidative stress [53,54], by scavenging toxins [53,55], and by supporting methylation processes [56]. Animals exposed to aspartame and its metabolites exhibited a range of problems, including increased levels of free radicals, oxidative stress, lipid peroxidation, inflammation, and mitochondrial dysfunction; increased permeability of the blood–brain barrier (BBB); excitotoxicity and neuronal apoptosis; and decreased brain serotonin, noradrenaline, and dopamine levels [25,26,27,29,38,41,42,44,45,46,50,51,57,58,59,60,61,62,63]. Several investigators attributed these adverse impacts to methanol and its metabolites [44,45,46,59,64].

Adverse impacts on the gut microbiota (GM) were also detected in animals exposed to aspartame [29,61,65,66,67,68,69,70,71,72,73] and two of its metabolites: formaldehyde [67] and phenylalanine [66]. Among other changes, aspartame-fed rats exhibited a doubling of serum propionate [65]. Propionic acid, a short-chain fatty acid produced by gut bacteria, was hypothesized to increase ASD risk through multiple pathways, including increased gut and BBB permeability [74,75]; decreased GSH and neurotransmitter levels; increased oxidative stress, excitotoxicity, and neuroinflammation [76,77]; and altered mitochondrial and immunological function [78].

**Figure 1:**
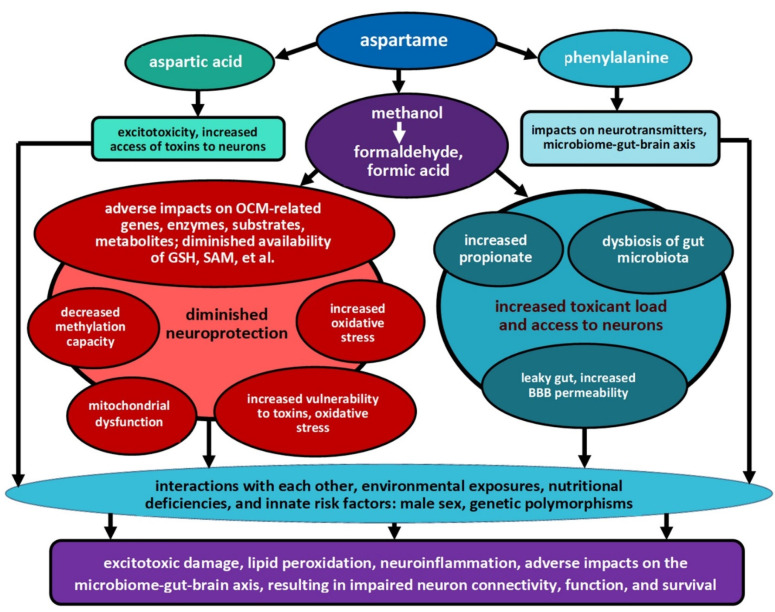
Potential impacts and interactions of aspartame and its metabolites on known autism risk factors. (OCM: one-carbon metabolism, including the transsulfuration pathway, methionine cycle, and folate cycle; GSH: reduced glutathione, a major antioxidant involved in defense against oxidative stress; detoxification; maintenance of methylation capacity; SAM: S-adenosylmethionine, the major methyl donor for cellular methylation processes; BBB: blood–brain barrier).

Taken together, as summarized in Figure 1, such physiological impacts could create a perfect storm: increasing the access of toxins to the developing brain, decreasing the developing brain’s antioxidant and detoxification capacities, and adversely impacting the microbiome–gut–brain axis, which is critical in neurodevelopment [79,80]. Further discussion of the processes summarized in Figure 1 is included in Section 4.

In order to address the question of whether maternal aspartame consumption increases autism risk in offspring exposed to aspartame in early life—in utero and during breastfeeding—we aimed to retrospectively assess the maternal intake during pregnancy and breastfeeding of diet soda and of aspartame from multiple sources, among biological mothers of offspring with either autism specifically or any ASD (cases) and among mothers of neurotypically developing offspring (controls), to evaluate the associations between daily early-life exposures to these products and current autism and ASD status in the case-control Autism Tooth Fairy Study (ATFS).

The primary aim of this study was to examine whether the odds of daily exposure during pregnancy/breastfeeding to ≥1 DS or an equivalent amount of aspartame, through the maternal diet, were significantly higher among offspring diagnosed with autism (autism cases), compared with neurotypically developing offspring (controls). The secondary aim of the study was to assess whether these exposure odds were higher among offspring with any ASD (ASD cases), compared with controls. Since males have increased developmental vulnerability to adverse early-life exposures and since the risk of autism is four times higher in boys than in girls, we performed both sex-specific and pooled analyses for both diagnostic categories.

## 2. Materials and Methods

### 2.1. Participants

Recruitment and data collection for the Autism Tooth Fairy Study were performed through the University of Texas Health Science Center at San Antonio (UTHSCSA) from May 2011 to June 2014. The largest recruitment source (*n* = 255) was the Interactive Autism Network (IAN), an Internet-based U.S. registry [81] that included >21,600 individuals with ASD and >22,000 parents of individuals with ASD [82]. Most families recruited through IAN had ≥1 child diagnosed with ASD (cases; *n* = 178). Neurotypically developing children (controls) identified through IAN recruitment included siblings of cases (*n* = 61), and offspring of friends/associates of IAN parents (*n* = 16) [83]. Additional recruitment (*n* = 101) was conducted through media outreach in San Antonio and South Texas (SA/STX), through which 57 cases, including 7 referred from local autism services, and 44 controls were enrolled. The ATFS, thus, included a total of 356 offspring: 235 ASD cases and 121 controls. In addition to completing questionnaires regarding their children’s early-life exposures, ATFS parents provided one or more of their children’s shed deciduous teeth for compositional analyses, results of which are reported elsewhere [84].

The study was conducted according to the guidelines of the Declaration of Helsinki and was approved by the Institutional Review Board (IRB) of the University of Texas Health Science Center at San Antonio (UTHSCSA) (UTHSCSA IRB protocol number 11-313, approved on 12 May 2011). In early data collection, all parents provided written informed consent before completing self-administered hard-copy questionnaires. During later Internet-based enrollment and data collection, all parents reviewed an IRB-approved online study information sheet prior to completing online questionnaires through secure websites, in accordance with the protocol approved by the IRB of the UTHSCSA.

### 2.2. Materials

#### 2.2.1. Demographic and Neurodevelopmental Data Collected

Parents provided demographic data about themselves and their households; the birth year and sex of each child; and whether each child had been diagnosed with either autism or autistic disorder, Asperger’s disorder, pervasive developmental disorder—not otherwise specified, or childhood disintegrative disorder. Children with any of these diagnoses were included as ASD cases. Parents were also asked whether each child had been diagnosed with any additional behavioral, developmental, and/or learning disabilities/disorders; non-cases with none of these diagnoses were included as controls.

Parents were asked, “Was there ever a time when your child used at least three words you could understand (besides mama or dada) on a daily basis for at least a month, and then seemed to stop talking for a while (at least a month) where he/she used no words that you could understand?” Cases where parents responded “no” were considered unlikely to have experienced a regressive form of autism or ASD and were, thus, categorized as “non-regressive” cases.

#### 2.2.2. Early-Life Exposures to Diet Sodas, Other Diet Drinks, Aspartame, and Other NNSs

Biological mothers completed retrospective questionnaires on their intake of diet sodas (DS) and other diet drinks (DD_other_) during pregnancy/breastfeeding. For each child they were asked, “While you were pregnant or breastfeeding your child, how often did you drink diet drinks containing artificial sweeteners? Please count diet sodas first, such as Diet Coke, Diet Dr. Pepper, and Diet Sprite, and then other diet drinks, such as Crystal Light, sugar-free Kool-Aid, Slim-Fast, and other ‘lite’ drinks”. Within each of these two beverage subcategories, mothers recorded the number of cans or bottles of DS and the number of glasses, cans, or bottles of DD_other_ that they had consumed per time unit; whether this time unit was daily, weekly, monthly, annually, or never; and the specific brand(s) consumed. Mean intake of DS/day and DD_other_/day was calculated and summed to estimate total maternal intake of diet drinks/day (DD_total_/day) during pregnancy/breastfeeding.

Each mother was also asked, “While you were pregnant or breastfeeding your child, how many little packets of low-calorie sweeteners (such as Sweet ‘N Low, Equal or Splenda) did you use in your coffee, tea, or other foods and beverages? In answering the question, please keep in mind the number of drinks you had each day, and how many packets of sweetener you added to each drink. Also, please include the number of packets you used in cereal or other food”. Intake of the following three leading NNS packets was specifically requested: “Equal/Nutrasweet (blue)”, “Splenda (yellow)”, and “Sweet’N Low (pink)”; space was left for recording intake of other NNS packet brands. Maternal estimates of intake of these products during pregnancy and breastfeeding were part of an extensive questionnaire that gathered data on maternal and child exposures to a number of household products and other environmental exposures throughout pregnancy and the early life of the child.

### 2.3. Procedure

#### 2.3.1. Case Definitions

Two case definitions were specified for analyses. The primary case definition of interest was autism disorder (autism); the secondary case definition was any ASD. In addition, subgroup analyses focused on offspring with non-regressive versions of each case definition, among whom the relative etiologic influence of intrauterine/neonatal exposures was expected to be maximized. In all analyses, controls were neurotypically developing offspring and—for sex-specific analyses—of the same sex as cases. Both sex-specific and pooled analyses were performed for each of the case definitions and exposures.

#### 2.3.2. Exposure Variables

Maternal intake of ≥12 ounces (1 can) of DS/day during pregnancy/breastfeeding defined the primary, dichotomized exposure variable: daily early-life exposure to DS (DS_early_). The secondary, dichotomized exposure variable—daily early-life exposure to aspartame (ASP_early_)—was defined as maternal intake of ≥177 mg/day of aspartame during pregnancy/breastfeeding, from the sum of aspartame dosages in DS + DD_other_ + packets consumed by the mother. This cut-point was selected because it equals the minimum aspartame content per can of the leading diet colas sweetened with aspartame alone. Estimates of total maternal aspartame intake from these three sources were possible because mothers were asked to report the specific brands they had consumed. Dosages of aspartame and other NNSs used in each of the leading brands of DS were previously published [85]; for comparable products by other brands, NNS dosages were imputed according to the NNS types specified on product labels. Leading DS brands exclusively sweetened with aspartame contained ≥177 mg of aspartame/can [85]; this dosage was, therefore, chosen as the cut-point for ASP_early_ to identify offspring with daily early-life aspartame exposure comparable to that in 1 can of the leading diet colas [85].

For DD_other_, ingredient-labeling-specified types of NNS used, but not dosages, which are proprietary. Based on limited available data, a 12-ounce serving of DD_other_ exclusively sweetened with aspartame was estimated to contain approximately 100 mg of aspartame. A tabletop packet contains 37 mg of aspartame [86].

### 2.4. Statistical Analyses

Offspring born in 1984 or thereafter, with complete early-life DS exposure data and neurodevelopmental diagnostic data, were included in analyses. The proportions of offspring with DS_early_ and ASP_early_ exposures were separately calculated, by sex, for the following diagnostic groups: controls, all ASD cases, ASD cases without autism, autism cases, and the non-regressive version of each case definition. Differences in the proportions of exposed controls and cases, in each category, were calculated using the Pearson χ^2^ statistic in Stata/IC 14.2 for Windows.

To assess associations among diagnostic status and the primary and secondary exposures under study—DS_early_ and ASP_early_—multilevel mixed-effects generalized linear models (MEGLMs) were used to consider potential influences at two levels: the individual child (level one) and shared intrauterine and familial influences, through the mother (level two). Recruitment source was treated as a level-one predictor. A total of 356 children, including children from 79 sibships, were included in analyses. Models were fit using the meglm command in Stata/IC 14.2 for Windows.

Unadjusted odds ratios (ORs) for the primary and secondary exposures of interest—DS_early_ and ASP_early_—were separately computed for each case definition, by sex, and for all participants, pooled. Adjusted models included the following covariates: recruitment source (IAN vs. SA/STX); year of birth (range, 1985 to 2011), to adjust for secular trends in autism diagnosis; and three dichotomous demographic variables: ethnicity of the child (non-Hispanic white (NHW) vs. other), maternal education (college graduate vs. not), and household income (≥USD 100,000/year vs. less). In pooled analyses, we additionally adjusted for sex of the child. For comparison purposes, we also calculated separate ORs for early-life exposures to ≥1 serving/day of aspartame specifically or to any NNS, from the combination of DS, DD_other_, and/or tabletop packets, with minimum serving size defined as one tabletop sweetener packet. The minimum daily dosage for these analyses would, thus, be 37 mg for aspartame, 36 mg for saccharin, and 12 mg for sucralose. We also performed subgroup analyses for non-regressive ASD and non-regressive autism, using these same exposure variables and covariates. These models were all fit using the meglm command in Stata/IC 14.2 for Windows.

### 2.5. Sensitivity Analyses

To examine influences on maternal intake of these products, we compared the proportions of biological mothers of males who reported consuming DS, aspartame, or any other NNSs during pregnancy and/or breastfeeding, within dichotomized substrata of three demographic factors previously associated with cardiometabolic risk: income, educational level, and ethnicity. Among these six demographic subsets—mothers of offspring from households with incomes ≥USD 100,000/year vs. less; mothers with ≥4 years of college vs. less education; and mothers of NHW children vs. children of other or mixed ethnicities—we compared the proportions of mothers whose mean total reported intake of diet products equaled or exceeded the dose of 1 packet/day of any NNS, 1 packet/day of aspartame, 1 DS/day, and 177 mg/day of aspartame. The significance of differences between sociodemographic substrata in the proportions of mothers who reported daily consumption of each of these diet products was assessed using the Pearson χ^2^ statistic in Stata/IC 14.2 for Windows.

## 3. Results

Table 1 displays characteristics of controls, ASD cases, autism cases, and non-regressive autism cases, by sex, for the 257 boys (203 ASD cases + 54 controls) and 99 girls (32 ASD cases + 67 controls) included in the analyses. Maternal education was high overall: ≥60% of mothers in each diagnostic/sex subgroup were college graduates. Over 60% of families had incomes above the 2010 U.S. median of USD 50,000 [87]; incomes were slightly higher for controls and non-regressive autism cases. Among boys, roughly 70% of both cases and controls were from IAN, and 70% of cases were NHW, although the proportion of controls who were NHW was slightly lower: 64%. Among girls, approximately 90% of cases were from IAN and were NHW, whereas approximately 60% of controls were from IAN and were NHW.

Among males, the proportion of offspring with daily DS_early_ and ASP_early_ exposures dramatically increased with the severity of the diagnostic category and further increased with the non-regressive condition (Table 2). Nearly one in four males with non-regressive autism (23.3%) had ASP_early_ exposures, and 22.1% had DS_early_ exposures, compared with 7.4% of controls. Among girls, no comparable trends emerged between their diagnostic status and either DS_early_ or ASP_early_ proportions.

Similarly, the exposure odds for DS_early_ progressively increased with diagnosis severity and non-regressive condition among males (Table 3). In the adjusted multivariable model, the exposure odds for DS_early_ were tripled among males with autism: OR = 3.14 (95% confidence interval (CI): 1.02–9.65) and were even higher for males with non-regressive autism: OR = 3.49 (95% CI: 1.10–11.1). No statistically significant associations were detected between DS_early_ and any of the following: total ASD in either sex; any ASD diagnostic category in pooled analyses; or any ASD diagnostic category in girls, among whom ORs were consistently <1. Thus, the association between DS_early_ and diagnostic status was specific to autism in males in our analyses.

Likewise, adjusted ORs for ASP_early_ progressively increased with diagnosis severity and non-regressive condition in males (Table 4), among whom ORs were more than tripled among all autism cases and even higher among non-regressive autism cases: OR = 3.42 (95% CI: 1.12–10.4), and OR = 3.72 (95% CI: 1.18–11.8), respectively. As in the case of DS_early_, no statistically significant associations were found between ASP_early_ and total ASD in boys, between ASP_early_ and any ASD-related diagnoses in girls (Table S1a), or among all participants combined (Table S1b). ORs for early-life exposure to ≥1 serving/day of any NNS—from either DS, DD_other_, or tabletop packets—increased with the severity of the diagnosis and ranged from 1.60 (95% CI: 0.69–3.70) for total ASD to 2.09 (95% CI: 0.84–5.18) for non-regressive autism, though were not statistically significant. ORs for daily early-life exposure to ≥1 serving/day of aspartame were higher and ranged from 1.94 (95% CI: 0.76–4.95) for total ASD to 2.63 (95% CI: 0.97–7.19) for non-regressive autism, but none of these was statistically significant. Taken together, these results suggest a possible threshold effect, with autism diagnosis in boys being associated with more-than-tripled odds of early-life exposure to either ≥1 DS/day or comparable daily aspartame exposure (≥177 mg/day) in this study. Lower daily dosages, however—for either NNS in general or aspartame specifically—were not associated with autism status in this study sample. Nor were statistically significant associations found between either DS_early_ or ASP_early_ exposure and total ASD in boys, between either exposure and any ASD diagnosis in girls (Table S1a), or among all participants combined (Table S1b).

### Sensitivity Analyses

The results from the sensitivity analyses for male offspring, displayed in Supplementary Figure S1a–c, demonstrate that the proportion of biological mothers consuming either ≥1 DS/day or ≥177 mg of aspartame/day were comparable across the substrata of household income, maternal education, and offspring ethnicity. However, point estimates for the proportions of mothers who reported consuming the mean equivalent of ≥1 packet/day of any NNS were slightly higher for offspring from more affluent vs. less affluent households, 32% vs. 25%, respectively (Figure S1a); and for offspring of mothers with college degrees vs. fewer years of education: 30% vs. 22%, respectively (Figure S1b). These proportions were comparable for NHWs and other ethnic groups: 27% vs. 26%, respectively (Figure S1c). None of the differences between the subgroups shown in Figure S1a–c were statistically significant. Among the mothers of all males in our study, 28% reported mean daily NNS consumption during pregnancy equivalent to ≥1 tabletop packet of NNS. These results are comparable to those from earlier studies, in which 24%–30% of pregnant women reported using DS/DSB or other NNSs during pregnancy [88,89,90].

## 4. Discussion

To our knowledge, this is the first study to specifically examine the associations between offspring autism status and the daily maternal intake of diet soda and aspartame during pregnancy/breastfeeding. Among boys, we found more than tripled odds of exposure to either DS_early_ or ASP_early_ among autism cases, compared with controls. These associations were both diagnosis- and sex-specific: no statistically significant associations were found for total ASD in boys nor for either diagnostic category in girls, who represented only 17% (*n* = 28) of total autism cases in our study.

Several possible explanations exist for the lack of associations among girls in our study; these include insufficient statistical power, inherent sex dimorphism in response to DS/aspartame exposures, and possibly even the recruitment strategy itself, which, by including as controls neurotypically developing female siblings of male cases, increased the likelihood that any early-life exposures found to be risk-enhancing among their brothers with ASD might appear to be negatively associated with ASD in the analyses for females. Further research with larger sample sizes for both sexes and prospectively gathered data would be important for investigating this association further in females and in all participants combined.

### 4.1. Non-Regressive Cases

Boterberg et al., in their overview of publications related to regression in ASD, noted that there is no single published definition for regression in ASD [91]. Along with Ozonoff et al. [92], however, they delineated language loss as a core component of regression in ASD [91,92] and noted that this component was included in the earliest descriptions of regression in autism [91]. Regression was historically observed to occur somewhere during the second year after birth or thereafter, although data from newer, prospective studies indicated that regression may occur at earlier ages, even within the first year after birth [91,92]. In an attempt to maximize our ability to detect associations between exposures in early life—in utero and/or through maternal milk—and current autism status and to minimize the influence of later-childhood exposures and influences on autism risk, we attempted to identify and focus on earlier-onset, non-regressive cases by designating a subset of cases whose parents had reported no loss of speech for them, at any point, as “non-regressive”. We then performed additional analyses for this subgroup, including only offspring without such reported language loss. Since, as Ozonoff et al. noted, regressive events may in fact also occur earlier in life without being detected [92], our “non-regressive” category may include some offspring with very-early-life regression; thus, the cases in this subgroup might best be broadly interpreted as “early-onset” and likely include both offspring whose differences were noted very early after birth and some for whom regression occurred somewhat later, though still earlier than the onset of speech acquisition.

We had hypothesized that, among these earlier-onset, “non-regressive” cases, the relative influence of prenatal exposures—compared with exposures later in infancy and early childhood—would be higher than that among all cases combined. Our findings supported this hypothesis: among males, the exposure ORs for non-regressive cases of ASD and autism routinely exceeded those for total ASD and autism cases.

### 4.2. Results from Earlier Studies in Adult Consumers and Gestationally Exposed Offspring

As previously noted, diet sodas and other diet beverages (DSB) are leading vehicles of aspartame intake in the U.S. [23]. Three clinical trials and two prospective studies previously reported adverse neurological reactions to aspartame and to the daily intake of diet beverages. Increased headache [18] and problems with nervousness/irritability, depression, memory, and spatial orientation [17,20] were reported among aspartame-consuming trial participants, with neurological symptomatology increased among individuals with a history of major depression [17]. Among 318,257 participants in the National Institutes of Health-American Association of Retired Persons (NIH-AARP) Diet and Health study followed for >10 years, incident depression was significantly higher among consumers of aspartame-sweetened tea and coffee, compared with non-consumers, which monotonically increased with DS consumption [21]. Similarly, among 1484 older members of the Framingham Heart Study Offspring cohort followed over a decade, the hazards of incident Alzheimer’s disease were nearly tripled among participants with cumulative DS consumption ≥ 1/day [22]. The increased incidence of other major cardiometabolic and related problems was also reported among daily adult DSB consumers [93,94,95].

Despite the adverse impacts from NNS and DSB consumption reported in studies of animals and human adults, respectively, the potential neurodevelopmental impacts of early-life exposure to NNSs in general and to DS/DSB in particular in humans remain largely unexplored. This is a question of particular concern because between 24% and 30% of pregnant women reported using either NNSs in general [88] or DS/DSB specifically [89,90] during their pregnancies. Furthermore, a recent Australian study reported detecting one or more NNSs in 100% of 15 cord blood samples analyzed and in 77% of 13 amniotic fluid samples analyzed [96]. Their study is the first to provide direct evidence of transplacental passage of NNSs in humans. Its authors also raised the troubling concern of the possible dose accumulation of NNSs within the fetus [96]. If early-life exposures to aspartame and one of its leading vehicles, DS, do indeed significantly increase autism risk in the unborn child, then it is critical for women of reproductive age to be informed of this association.

Health impacts among the very young—a particularly vulnerable population—remained largely unstudied in humans until 2010, when a publication from the first of three prospective studies described an increased risk of adverse health outcomes among offspring exposed daily in utero to DSB [64,89,97,98] or to the combination of DS + aspartame packets [99]. These studies were the Danish National Birth Cohort (DNBC; *n* = 60,466 pregnant women and their infants) [64,97]; the Canadian Healthy Infant Longitudinal Development Study (CHILD; *n* = 2686 pregnant women and their infants) [89,98]; and Project Viva (PV; *n* = 1683 children from a prospective pre-birth cohort) [99]. In the DNBC, daily maternal DSB intake during pregnancy was associated with a 38% increase in total preterm birth (<37 weeks’ gestation); a 67% increase in early-preterm birth (<32 weeks’ gestation) [64]; and—among mothers with gestational diabetes—almost doubled risk of offspring overweight/obesity by age 7 years [97]. Similarly, offspring overweight/obesity by age 1 year was doubled among CHILD offspring exposed to DSB daily in utero [89]. In Project Viva, the children of mothers in the highest quartile of DS + aspartame consumption during pregnancy had significantly higher BMI z-scores and sum of skinfolds throughout early and middle childhood, compared with unexposed offspring [99]. Furthermore, the association between maternal NNS intake during pregnancy and subsequent offspring’s BMI *z*-scores increased from age 3 to 18 years [99]. Thus, in each of these prospective studies, daily gestational exposure to these products was associated with increased cardiometabolic risk among offspring, even after adjustment for such important risk factors as pre-pregnancy maternal body mass index, caloric intake, physical activity, diabetes status, and demographic characteristics.

### 4.3. Sex Differences in Developmental Responses to Early-Life Exposures from Earlier Studies

In the results from both CHILD [89] and the DNBC [97], it is notable that the doubling of the overweight/obesity risk observed among DSB-exposed offspring occurred in male, but not in female, offspring [89,97]. This is of particular relevance to the current investigation because increased neurodevelopmental vulnerability among males was previously reported for both intrauterine and other early-life exposures, including environmental neurotoxicants [100], maternal stress [101], and dysbiosis of the gut microbiota [79]. This heightened vulnerability among males contributes to the four- to eight-fold increased risk of neurodevelopmental problems in human males [100,101].

### 4.4. Is Dietary Methanol from Aspartame Contributing to These Associations?

As noted earlier, the DNBC previously reported a 38% increased risk of total preterm birth (<37 weeks’ gestation), and a 67% increased risk of early-preterm birth (<32 weeks’ gestation) among offspring whose mothers had consumed DSB daily during their gestation [64]; the authors suggested that the aspartame metabolite methanol may have contributed to these impacts. Gestational exposures to dietary methanol were estimated in an earlier study by Walton and Monte [7] for 161 ASD cases and 550 controls, by means of retrospective dietary questionnaires completed by their biological mothers. The mothers reported their intake of key dietary sources of methanol—including DS, other diet products, and processed fruit and vegetable juices—during pregnancy. Based on their estimates, the mean calculated dietary methanol exposure in utero for ASD cases was more than double that among controls: 142 vs. 67 mg/day, respectively, for both sexes combined (*p* < 0.001 for difference) [7]. Although the key exposure of interest in their study and the range and specificity of the dietary products examined differed from ours, Walton and Monte were the first to report an association between estimated gestational dietary exposure to an aspartame metabolite—methanol—and subsequent ASD status. The congruence of their findings with our own offers support for the need to further investigate the influence of these early-life dietary exposures on autism risk.

### 4.5. Metabolic Impacts of Aspartame, Methanol, and Their Metabolites on Availability of Reduced Glutathione (GSH) and Other Products of One-Carbon Metabolism

One of the most frequently reported impacts of aspartame consumption in animal studies is a decrease in the availability of GSH, as well as the adverse metabolic impacts that follow from it. GSH and S-adenosyl methionine (SAM), the major methyl donor for cellular methylation processes [52], are among a number of neuroprotective molecules maintained through folate-dependent one-carbon metabolism (OCM), a system of interrelated processes that include the folate cycle, the methionine cycle, and the transsulfuration pathway, through which GSH is synthesized, and redox balance is maintained [102]. Dysregulation of OCM was observed in both aspartame-fed animals and individuals with autism [49,50,52,103,104,105]. In mice, the aspartame-triggered blockade of the transsulfuration pathway and the methionine cycle within OCM has led to the reduced availability of GSH, SAM, and other OCM metabolites [52]. It is notable that decreased GSH and increased oxidative stress, mitochondrial dysfunction, inflammation, dysbiosis of the gut microbiota, and leaky gut were also observed in both animals fed aspartame and individuals with autism [75,103,106,107,108].

### 4.6. Sensitivity Analyses

Since maternal overweight and obesity (OW/OB), diabetes, and related conditions are associated with an increased risk of ASD among offspring [109,110,111,112,113,114] and might also influence a woman’s decision to use diet products, these represent potential unmeasured confounders in our results. Although we have no data on maternal cardiometabolic risk factors in our study sample, we have data for three key demographic measures—household income, educational attainment, and ethnicity—which were previously associated with cardiometabolic risk in population-based studies. In earlier studies, a higher prevalence of both OW/OB and diabetes was found among individuals with lower education and income [115,116,117] and in some ethnic groups other than NHWs [118,119]. We, therefore, included these three sociodemographic measures in all adjusted calculations of ORs for DS_early_ and ASP_early_ (reported in Table 3, Table 4 and Table S1a,b). In addition, for males, we compared the maternal consumption of different diet products within demographic subgroups defined by these variables.

A higher consumption of diet products within demographic subgroups historically at a higher risk of OW/OB and diabetes would be expected if maternal consumption of diet products in our study population was primarily driven by pre-existing cardiometabolic problems. Our sensitivity analyses (Figure S1a–c), however, revealed that the proportion of mothers with average NNS intakes ≥ 1 packet/day of NNS were similar across ethnic subgroups (Figure S1c) and were in fact higher among mothers with college degrees (Figure S1b) and from more affluent households (Figure S1a), although none of these differences were statistically significant. These results were congruent with those from the National Health and Nutrition Examination Survey (NHANES) data from 1999–2000 to 2013–2014, in finding NNS consumption to be higher among NHWs and among participants with higher education and income [88]. The NHANES data, like our own, suggest the possibility that NNS consumption may have been primarily adopted as a preventive, health-promotion strategy, rather than simply in response to existing cardiometabolic problems, in each of these study populations.

### 4.7. Future Directions

While our findings do not establish a causal relationship between daily early-life exposure to diet sodas/aspartame and autism risk in males, they nonetheless raise concerns that justify further research, especially given the current widespread use of diet products among pregnant women. It would be important to study the association between maternal DBS/NNS intake during pregnancy and subsequent offspring autism risk within the context of a larger population in which both the maternal intake of these products and additional maternal characteristics, including cardiometabolic and other risk factors, could be prospectively measured. In the meantime, the congruence between our results and those of earlier studies in finding associations between early-life exposures to DSB and/or aspartame and subsequent chronic health problems in offspring suggests a pattern of increased risk that requires further study.

Autism typically results from a perfect storm of adverse impacts, with toxicant exposures overlaid upon genetic polymorphisms that confer decreased neuroprotection [102]. As previously summarized, early-life aspartame exposure could potentially contribute to this storm by increasing toxicant load while simultaneously decreasing neuroprotective potential, as indicated in Figure 1. Although daily early-life exposure to DS/aspartame represents only one potential risk-increasing exposure within the total exposome of the developing child, nonetheless—within the context of male sex and other risk factors—it might contribute to overwhelming the delicate neurodevelopmental susceptibility tipping point of a child already at increased risk.

### 4.8. Strengths and Limitations

Our dietary questionnaires were retrospectively completed and focused on maternal intake several years earlier, during pregnancy/breastfeeding. Diagnostic status, elapsed time, and/or changes in product formulations may have led to recall bias and/or other information bias in the maternal dietary recalls. As noted earlier, no covariate data were available for maternal overweight/obesity and diabetes, maternal mental health, and other potential confounders in our study. In addition, no dietary recall data were available for the paternal intake of these products during the preconceptual period. Our estimates of offspring aspartame exposure were based upon the maternal intake of three product categories: diet sodas, other diet beverages, and sweetener packets. While these are prominent vehicles for aspartame consumption, aspartame itself is included as an ingredient in over 6000 dietary, pharmacologic, and cosmetic products [8]; thus, our intake estimates only represented the minimum maternal intake of aspartame: both cases and controls were likely exposed to aspartame from additional sources. The overall study sample was small and had low statistical power. In addition, the number of controls relative to cases was small, especially among males, and included both unrelated controls and siblings of cases. Further research is needed to determine whether the apparent associations observed in our study are artifacts of these limitations or in fact arise from the deleterious consequences of early-life exposures to these products. In support of a causal relationship between these early-life exposures and autism risk in males is the striking congruence between our results and those from animal studies as well as from large-scale prospective studies that—even after adjusting for both maternal body mass index, diabetes status, and other key potential confounders—found striking associations between daily gestational exposure to non-nutritive sweeteners and/or diet beverages and subsequent major health problems among offspring, especially among males.

## 5. Conclusions

Compared with male controls, males with autism in our study had more than tripled odds of having been exposed daily—gestationally and/or through breastfeeding—to either diet soda itself or comparable doses of aspartame from multiple sources. These exposure odds were the highest among cases with non-regressive autism. These associations do not prove causality. Taken in concert, however, with previous findings of increased prematurity and cardiometabolic health impacts among infants and children exposed daily to diet beverages and/or aspartame during pregnancy, they raise new concerns about the potential neurological impacts, which need to be addressed. Further research—including larger sample sizes of both sexes and prospective measurement of exposures, additional potential confounders, and outcomes—is needed to evaluate these associations in other study populations and to assess whether they extend to overall ASD risk in boys and to autism and/or ASD risk in girls.

In the meantime, however, the possibility that early-life exposures to these products through maternal diet might increase offspring neurodevelopmental risk—at least among boys—is of particular concern. Between 24% and 30% of pregnant women reported using either non-nutritive sweeteners in general [88] or diet sodas and other diet beverages specifically [89,90] during their pregnancies. Furthermore, a recent study provided evidence of the transplacental passage of these substances in humans [96] and raised troubling questions regarding the dose accumulation of non-nutritive sweeteners within the fetus. Taken together with widespread gestational exposure to these products and previously reported associations between gestational exposure to these products and adverse health impacts in offspring, these findings suggest that, in the spirit of the Precautionary Principle [120], women should exercise caution when considering the use of these products during pregnancy and breastfeeding, until further assessments are available. The maternal consumption of these products during periods of heightened offspring vulnerability represents a modifiable potential risk factor, the elimination of which might help to protect susceptible offspring in the next generation.

## Figures and Tables

**Table 1:** Demographic characteristics, by sex and diagnostic status, among 356 offspring in the Autism Tooth Fairy Study.

Characteristic	Boys (*n* = 257)				Girls (*n* = 99)			
		Cases				Cases		
	Controls (*n* = 54)	Any ASD (*n* = 203)	Autism (*n* = 140)	Non-Regressive Autism (*n* = 86)	Controls (*n* = 67)	Any ASD (*n* = 32)	Autism (*n* = 28)	Non-Regressive Autism (*n* = 19)
**Birth year of child** **(Mean (SD))**	**2001.3** (4.1)	**2001.4** (4.3)	**2001.5** (4.2)	**2001.5** (4.1)	**2002.2** (3.8)	**2001.8** (4.3)	**2001.9** (4.3)	**2001.4** (4.6)
**Education of mother (*n*)**	**54**	**202**	**139**	**86**	**67**	**32**	**28**	**19**
<High school (%)	0	4	3	3	3	0	0	0
High school graduate (%)	13	5	8	9	4	9	7	5
<4 yrs college (%)	20	31	29	19	28	25	29	21
≥4 yrs college (%)	67	60	60	69	64	66	64	74
**Family income (*n*)**	**50**	**198**	**135**	**83**	**66**	**32**	**28**	**19**
<USD 25K (%)	12	18	19	16	14	9	7	5
USD 25K to <USD 50K (%)	10	16	14	12	17	28	29	16
USD 50K to <USD 100K (%)	32	35	36	30	29	34	39	53
USD 100K to <USD 200K (%)	32	25	25	34	29	19	18	21
≥USD 200K (%)	14	7	7	8	12	9	7	5
**Ethnicity of child (*n*)**	**53**	**202**	**139**	**85**	**66**	**32**	**28**	**19**
Hispanic (%)	25	16	15	13	32	9	7	5
Non-Hispanic white (%)	64	70	68	74	58	88	93	95
Other/mixed (%)	11	14	17	13	11	3	0	0
**Recruitment source (*n*)**	**54**	**203**	**140**	**86**	**67**	**32**	**28**	**19**
IAN (%)	69	74	73	72	60	87	89	89
SA/STX (%)	31	26	27	28	40	13	11	11

ASD = autism spectrum disorder; SD = standard deviation; IAN = Interactive Autism Network; SA/STX = San Antonio/South Texas; K = 1000.

**Table 2:** Percentage of cases and controls exposed to ≥1 diet soda or ≥177 mg of aspartame daily ^a^.

		Boys (*n* = 257)			Girls (*n* = 99)	
	*n*	≥1 Diet Soda/Day	≥177 mg/Day of Aspartame	*n*	≥1 Diet Soda/Day	≥177 mg/Day of Aspartame
		%	%		%	%
**Controls**	54	7.4	7.4	67	17.9	19.4
**ASD cases, excluding autism**	63	9.5	9.5	4	25.0	25.0
Non-regressive ASD cases, excluding autism	45	11.1	11.1	4	25.0	25.0
**All ASD cases, combined**	203	16.3	17.2	32	12.5	12.5
Non-regressive ASD cases, combined	131	18.3	19.1 *	23	17.4	17.4
**Autism cases**	140	19.3 *	20.7 *	28	10.7	10.7
Non-regressive autism cases	86	22.1 *	23.3 *	19	15.8	15.8

^a^ In early life and during gestation and/or breastfeeding; based on dietary intakes retrospectively reported by biological mothers. Bold-highlighted results indicate statistically significantly increased exposure proportions among cases, compared with controls. * *p* < 0.05 for difference from proportion of male controls with daily early-life exposures to these products.

**Table 3:** Odds ratios ^a^ (OR) for early-life exposure ^b^ to ≥1 diet soda/day, by autism spectrum disorder (ASD) category.

Condition	Boys			Girls			All Participants Combined ^c^		
	*n*	OR	95% CI	*n*	OR	95% CI	*n*	OR	95% CI
**ASD: all cases**									
Unadjusted ^c^	257	2.4	0.8 to 7.2	99	0.7	0.2 to 2.2	356	1.4	0.7 to 2.8
Adjusted ^d^	246	2.4	0.8 to 7.3	98	0.5	0.1 to 1.8	344	1.4	0.7 to 2.9
**Non-regressive ASD**									
Unadjusted ^c^	185	2.8	0.9 to 8.5	90	1.0	0.3 to 3.4	275	1.8	0.8 to 3.8
Adjusted ^d^	177	2.7	0.9 to 8.4	89	0.7	0.2 to 3.0	266	1.8	0.8 to 3.8
**Autism**									
Unadjusted ^c^	194	3.0	0.99 to 9.0	95	0.6	0.1 to 2.1	289	1.5	0.7 to 3.1
Adjusted ^d^	183	3.1 *	**1.02 to 9.7**	94	0.3	0.1 to 1.3	277	1.5	0.7 to 3.2
**Non-regressive autism**									
Unadjusted ^c^	140	3.5 *	**1.1 to 11.1**	86	0.9	0.2 to 3.4	226	2.0	0.9 to 4.3
Adjusted ^d^	132	3.5 *	**1.1 to 11.1**	85	0.5	0.1 to 2.3	217	2.0	0.9 to 4.4

^a^ ORs were derived using multilevel mixed-effects generalized linear model analyses. Bold-highlighted results indicate statistically significantly increased exposure ORs (*p* < 0.05). ^b^ Early-life exposure: through maternal diet during pregnancy and breastfeeding. Exposures were calculated based on diet soda intake during this period, retrospectively recalled by biological mothers, for 356 offspring in the Autism Tooth Fairy Study. ^c^ Sex was included as a covariate in both “unadjusted” and adjusted analyses for all participants combined. ^d^ Adjusted for mother’s identity; recruitment source; child’s ethnicity (non-Hispanic white vs. other); year of birth; mother’s education (≥4 years of college vs. less), and household income (≥USD 100,000/year vs. less). OR = odds ratio; CI = confidence interval; * *p* < 0.05 for difference from odds of daily early-life exposure to ≥1 diet soda, among controls.

**Table 4:** Adjusted odds ratios ^a^ among males for daily early-life ^b^ exposures to any NNS ^c^ or to aspartame specifically ^d^.

Condition in Males	*n*	Daily Exposure	ORs	95% CI
**ASD: all cases**	246	≥1 serving/day of any NNS	1.6	0.7 to 3.7
		≥1 serving/day of aspartame	1.9	0.8 to 5.0
		≥177 mg/day of aspartame	2.6	0.9 to 7.8
**Non-regressive ASD**	177	≥1 serving/day of any NNS	1.7	0.7 to 4.1
		≥1 serving/day of aspartame	2.1	0.8 to 5.7
		≥177 mg/day of aspartame	2.9	0.9 to 8.8
**Autism**	183	≥1 serving/day of any NNS	2.0	0.8 to 4.7
		≥1 serving/day of aspartame	2.5	0.95 to 6.5
		**≥177 mg/day of aspartame**	3.4 *	**1.1 to 10.4**
**Non-regressive autism**	132	≥1 serving/day of any NNS	2.1	0.8 to 5.2
		≥1 serving/day of aspartame	2.6	0.97 to 7.2
		**≥177 mg/day of aspartame**	3.7 *	**1.2 to 11.8**

^a^ ORs were derived using multilevel mixed-effects generalized linear model analyses for 246 male offspring in the Autism Tooth Fairy Study: adjusted for mother’s identity; recruitment source; child’s ethnicity (non-Hispanic white vs. other); year of birth; mother’s education (≥4 years of college vs. less), and household income (≥USD 100,000/year vs. less). Bold-highlighted results indicate statistically significantly increased exposure ORs (*p* < 0.05). ^b^ Early-life exposures: exposures that occurred during gestation and/or breastfeeding, through maternal diet during these times. These were calculated based on aspartame and other NNS intake during this period, from the sum of diet sodas, other diet drinks, and sweetener packets, retrospectively recalled by biological mothers. ^c^ NNS: non-nutritive sweetener: ≥1 serving/day of any NNS denotes either ≥1 packet/day of any NNS, ≥1 DS/day, or ≥1 other diet drink/day. Minimum daily dosage for this category, thus, varies by NNS: 1 tabletop packet contains 36 mg of saccharin, 12 mg of sucralose, or 37 mg of aspartame, for example. ^d^ For aspartame specifically: ≥1 serving/day of aspartame denotes either ≥1 packet/day of aspartame, ≥1 aspartame-sweetened DS/day, or ≥1 other aspartame-sweetened diet drink/day. Minimum daily dosage for this category is, thus, 37 mg, the dosage in 1 tabletop packet. ≥177 mg/day of aspartame denotes total daily aspartame intake, from the sum of packets + DS + other diet drinks, equivalent to ≥177 mg, the dosage of aspartame in 1 can of a leading diet cola sweetened only with aspartame. * *p* < 0.05 for difference from odds of daily early-life exposure to ≥177 mg of aspartame, among male controls.

## Data Availability

The data presented in this study are openly available in the Dryad Digital Repository at https://doi.org/10.5061/dryad.cfxpnvx2p [121].

## Supplementary Materials

The following supporting information can be downloaded at https://www.mdpi.com/article/10.3390/nu15173772/s1. Table S1a: Adjusted ORs among female offspring for daily early-life exposures to any NNS or to aspartame specifically; Table S1b: Adjusted ORs among all offspring for daily early-life exposures to any NNS or to aspartame specifically; Figure S1a: Among mothers of all males, percentage who consumed ≥1 DS/day, ≥177 mg/day of aspartame, ≥1 packet/day of aspartame (mean daily equivalent), or ≥1 packet/day of any NNS (mean daily equivalent) during pregnancy and/or breastfeeding, by substratum of household income; Figure S1b: Among mothers of all males, percentage who consumed ≥1 DS/day, ≥177 mg/day of aspartame, ≥1 packet/day of aspartame (mean daily equivalent), or ≥1 packet/day of any NNS (mean daily equivalent) during pregnancy and/or breastfeeding, by substratum of maternal education; Figure S1c: Among mothers of all males, percentage who consumed ≥1 DS/day, ≥177 mg/day of aspartame, ≥1 packet/day of aspartame (mean daily equivalent), or ≥1 packet/day of any NNS (mean daily equivalent) during pregnancy and/or breastfeeding, by ethnic group.
