# Supplementary material for: Daily Early-Life Exposures to Diet Soda and Aspartame Are Associated with Autism in Males: A Case-Control Study
Source: Nutrients. 2023 Aug 29;15(17):3772. doi: 10.3390/nu15173772 (PMC10490529; doi:10.3390/nu15173772)
Supplement: Supplementary file 1 [file nutrients-15-03772-s001.zip › Supplemental Table S1a.pdf]

**Supplemental Table S1a: Adjusted odds ratios[a] (ORs) among female offspring for daily early-life[b] exposures to any NNS[c] or to aspartame specifically[d]**

| Condition in males | n | Daily exposure | ORs | 95% CI |
|---|---|---|---|---|
| **ASD: all cases** | 98 | ≥1 serving/day of any NNS | 0.70 | 0.22 to 2.22 |
| | | ≥1 serving/day of aspartame | 0.44 | 0.11 to 1.71 |
| | | ≥177 mg/day of aspartame | 0.45 | 0.11 to 1.77 |
| **Non-regressive ASD** | 89 | ≥1 serving/day of any NNS | 1.13 | 0.35 to 3.70 |
| | | ≥1 serving/day of aspartame | 0.70 | 0.17 to 2.80 |
| | | ≥177 mg/day of aspartame | 0.71 | 0.18 to 2.87 |
| **Autism** | 94 | ≥1 serving/day of any NNS | 0.54 | 0.15 to 1.92 |
| | | ≥1 serving/day of aspartame | 0.26 | 0.05 to 1.25 |
| | | ≥177 mg/day of aspartame | 0.26 | 0.05 to 1.27 |
| **Non-regressive autism** | 85 | ≥1 serving/day of any NNS | 0.95 | 0.25 to 3.57 |
| | | ≥1 serving/day of aspartame | 0.45 | 0.09 to 2.26 |
| | | ≥177 mg/day of aspartame | 0.45 | 0.09 to 2.28 |

[a] Among 98 female offspring in the Autism Tooth Fairy Study, adjusted for mother's id; recruitment source; child's ethnicity (non-Hispanic white vs. other); year of birth; mother's education (≥4 years of college, vs. less), and household income (≥$100,000/year vs. less).

[b] Early-life exposures: exposures that occurred during gestation and/or breastfeeding, through maternal diet during these times. These were calculated based on aspartame and other NNS intake during this period, retrospectively recalled by biological mothers.

[c] NNS: non-nutritive sweetener:

≥1 serving/day of any NNS denotes either ≥1 packet/day of any NNS, ≥1 DS/day, or ≥1 other diet drink/day. Minimum daily dosage for this category thus varies by NNS: 1 tabletop packet contains 36 mg of saccharin, 12 mg of sucralose, or 37 mg of aspartame.

[d] For aspartame specifically:

≥1 serving/day of aspartame denotes either ≥1 packet/day of aspartame, or ≥1 aspartame-sweetened DS/day, or ≥1 other aspartame-sweetened diet drink/day. Minimum daily dosage for this category is thus 37 mg of aspartame, the dosage in 1 tabletop packet.

≥177 mg/day of aspartame denotes total daily aspartame intake, from the sum of packets + DS + other diet drinks, equivalent to 177 mg, the dosage of aspartame in 1 can of a leading diet cola sweetened only with aspartame.

OR: odds ratio; NNS: non-nutritive sweetener; CI: confidence interval; ASD: autism spectrum disorder
