# Supplementary material for: Daily Early-Life Exposures to Diet Soda and Aspartame Are Associated with Autism in Males: A Case-Control Study
Source: Nutrients. 2023 Aug 29;15(17):3772. doi: 10.3390/nu15173772 (PMC10490529; doi:10.3390/nu15173772)
Supplement: Supplementary file 1 [file nutrients-15-03772-s001.zip › Supplemental Table S1b.pdf]

Supplemental Table S1b: Adjusted odds ratios[a] (ORs) among all offspring for daily early-life[b] exposures to any NNS[c] or to aspartame specifically[d]

Condition in males	n	Daily exposure	ORs	95% CI
**ASD: all cases**	344	≥1 serving/day of any NNS	1.23	0.66 to 2.31
		≥1 serving/day of aspartame	1.19	0.61 to 2.33
		≥177 mg/day of aspartame	1.39	0.68 to 2.84
**Non-regressive ASD**	266	≥1 serving/day of any NNS	1.53	0.78 to 2.99
		≥1 serving/day of aspartame	1.51	0.74 to 3.07
		≥177 mg/day of aspartame	1.77	0.83 to 3.77
**Autism**	277	≥1 serving/day of any NNS	1.38	0.73 to 2.64
		≥1 serving/day of aspartame	1.33	0.67 to 2.66
		≥177 mg/day of aspartame	1.58	0.76 to 3.26
**Non-regressive autism**	217	≥1 serving/day of any NNS	1.76	0.87 to 3.54
		≥1 serving/day of aspartame	1.68	0.80 to 3.53
		≥177 mg/day of aspartame	2.03	0.93 to 4.44

[a] Among all 344 offspring in the Autism Tooth Fairy Study, adjusted for mother's id; recruitment source; child's ethnicity (non-Hispanic white vs. other); year of birth; mother's education (≥4 years of college, vs. less), and household income (≥$100,000/year vs. less).

[b] Early-life exposures: exposures that occurred during gestation and/or breastfeeding, through maternal diet during these times. These were calculated based on aspartame and other NNS intake during this period, retrospectively recalled by biological mothers.

[c] NNS: non-nutritive sweetener:

≥1 serving/day of any NNS denotes either ≥1 packet/day of any NNS, ≥1 DS/day, or ≥1 other diet drink/day. Minimum daily dosage for this category thus varies by NNS: 1 tabletop packet contains 36 mg of saccharin, 12 mg of sucralose, or 37 mg of aspartame, for example.

[d] For aspartame specifically:

≥1 serving/day of aspartame denotes either ≥1 packet/day of aspartame, or ≥1 aspartame-sweetened DS/day, or ≥1 other aspartame-sweetened diet drink/day. Minimum daily dosage for this category is thus 37 mg of aspartame, the dosage in 1 tabletop packet.

≥177 mg/day of aspartame denotes total daily aspartame intake, from the sum of packets + DS + other diet drinks, equivalent to 177 mg, the dosage of aspartame in 1 can of a leading diet cola sweetened only with aspartame.

Bold-highlighted results indicate statistically significantly increased exposure ORs ($p<0.05$).

OR: odds ratio; NNS: non-nutritive sweetener; CI: confidence interval; ASD: autism spectrum disorder
