# Supplementary figures and images for: Daily Early-Life Exposures to Diet Soda and Aspartame Are Associated with Autism in Males: A Case-Control Study
Source: Nutrients. 2023 Aug 29;15(17):3772. doi: 10.3390/nu15173772 (PMC10490529; doi:10.3390/nu15173772)

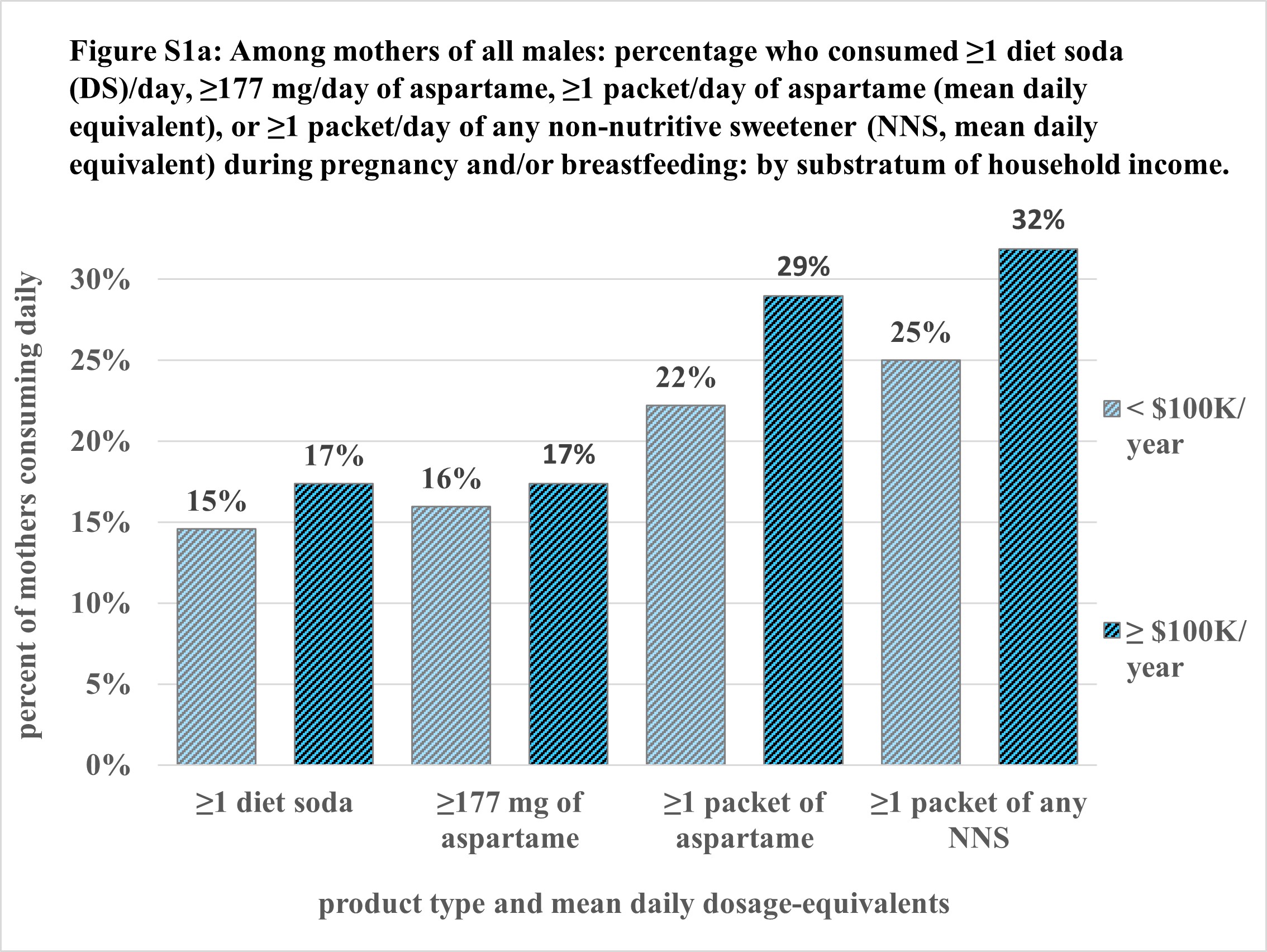

Supplement: Supplementary file 1 [file nutrients-15-03772-s001.zip › Figure S1a.jpg]

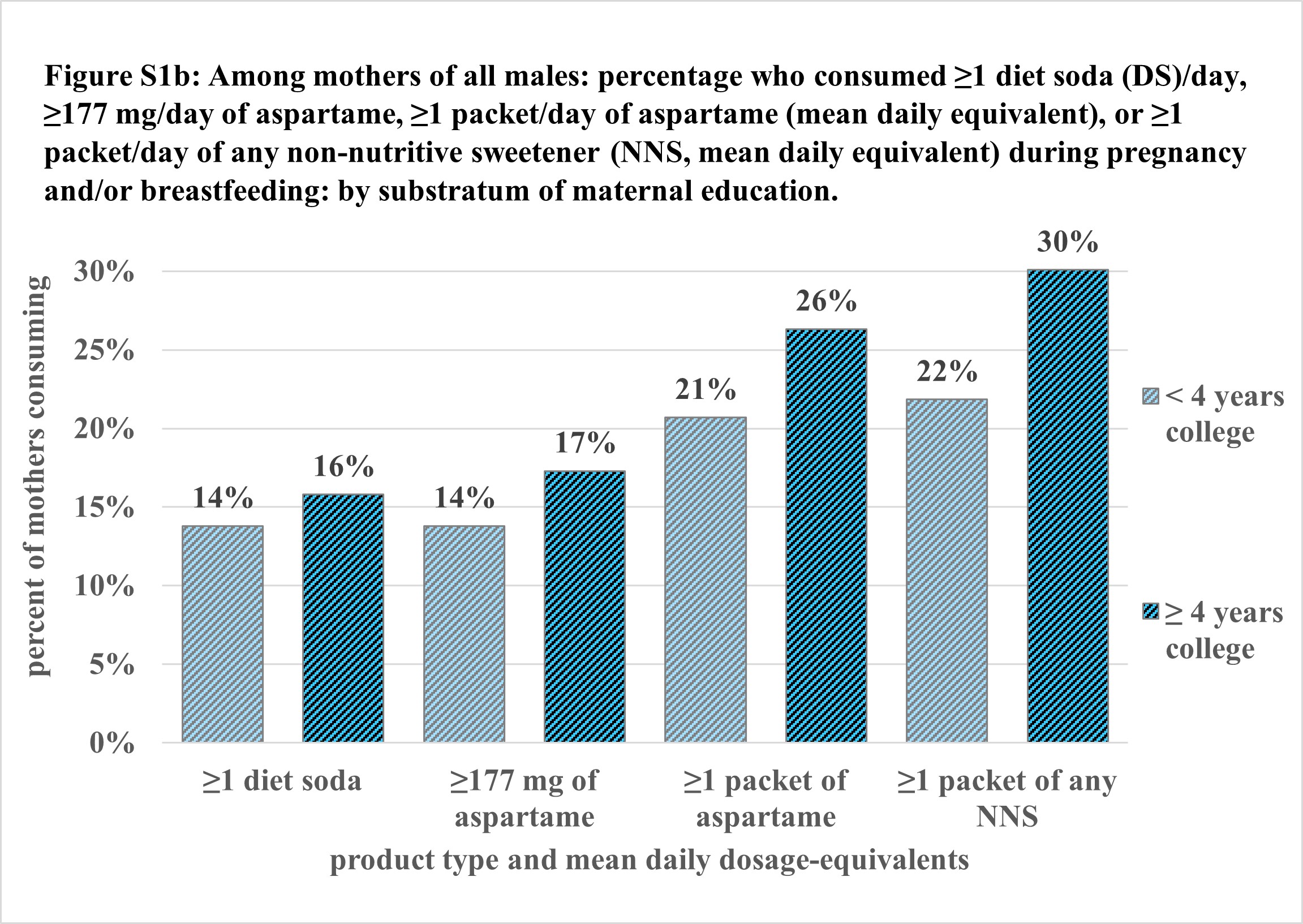

Supplement: Supplementary file 1 [file nutrients-15-03772-s001.zip › Figure S1b.jpg]

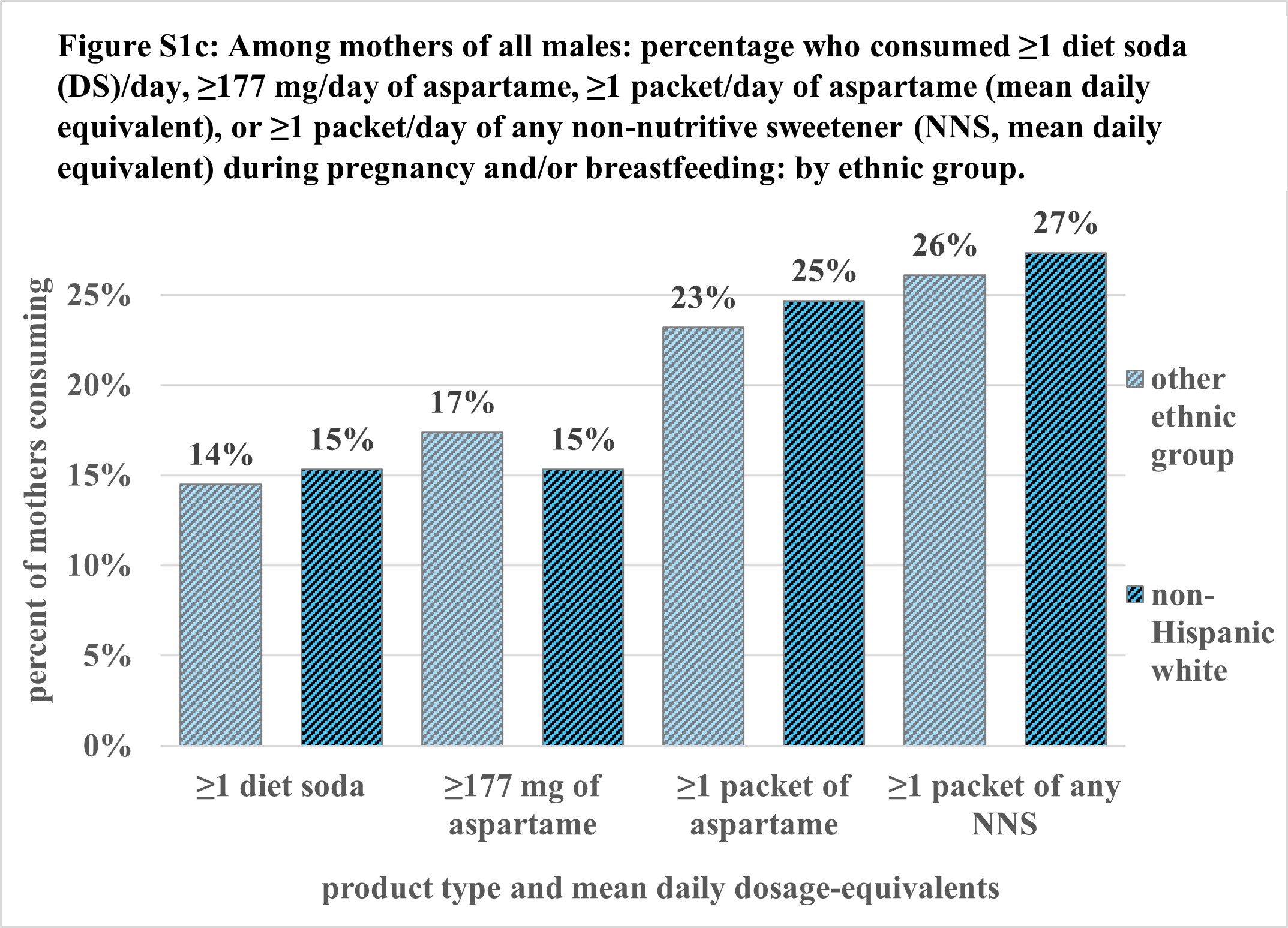

Supplement: Supplementary file 1 [file nutrients-15-03772-s001.zip › Figure S1c.jpg]
